# Immunity and Breast Cancer: Focus on Eosinophils

**DOI:** 10.3390/biomedicines9091087

**Published:** 2021-08-26

**Authors:** Aurélie Poncin, Concetta Elisa Onesti, Claire Josse, Delphine Boulet, Jérôme Thiry, Vincent Bours, Guy Jerusalem

**Affiliations:** 1 Department of Medical Oncology, University Hospital of Liege, CHU Sart Tilman, 4000 Liege, Belgium; aponcin@chuliege.be (A.P.); g.jerusalem@chuliege.be (G.J.); 2 Clinical and Oncological Research Department, IRCCS Regina Elena National Cancer Institute, 00144 Rome, Italy; 3 Laboratory of Human Genetics, GIGA Research Center, University of Liège, 4000 Liege, Belgium; c.josse@chuliege.be (C.J.); dboulet.pro@gmail.com (D.B.); j.thiry@uliege.be (J.T.); vbours@ulg.ac.be (V.B.); 4 Department of Medical Oncology, University of Liege, 4000 Liege, Belgium

**Keywords:** breast cancer, immunity, eosinophils, immunosurveillance

## Abstract

The role of eosinophils, a cell type involved in the immune response to parasitic infections and allergies, has been investigated in different cancer types, in both tumor tissue and at the circulating level. Most studies showed a role mainly in conjunction with immunotherapy in melanomas and lung tumors, while few data are available in breast cancer. In this review, we summarize literature data on breast cancer, showing a prognostic role of circulating eosinophil counts as well as of the presence of tumor tissue infiltration by eosinophils. In particular, some studies showed an association between a higher circulating eosinophil count and a good prognosis, as well as an association with response to neoadjuvant chemotherapy in hormone receptor-negative/HER2-positive and in triple negative breast cancer. Several mechanistic studies have also been conducted in in vivo models, but the exact mechanism by which eosinophils act in the presence of breast cancer is still unknown. Further studies on this subject are desirable, in order to understand their role at the cellular level, identify related biomarkers and/or possibly search for new therapeutic targets.

## 1. Introduction

Breast cancer is the most common cancer and the leading cause of cancer-related death in women worldwide, with more than 2 million new cases and more than 600,000 deaths per year [1]. Early breast cancer is usually treated with surgery, radiotherapy and systemic therapy (endocrine therapy, chemotherapy, targeted therapy) depending on prognostic and predictive factors (endocrine receptors, HER2 amplification). Advanced breast cancer is treated with endocrine therapy, targeted therapy or chemotherapy according to patient and cancer characteristics and line of treatment. Immunotherapy has an emerging role in breast cancer with two drugs, Atezolizumab and Pembrolizumab, recently approved by the FDA, and Durvalumab shown to improve outcomes in the neoadjuvant setting in triple negative breast cancer (TNBC) in the GeparNUEVO trial, presented during the 2021 ASCO congress [2].

The immune system plays an important role in cancer development and progression [3]. Recent reports also suggest a role of the immune system in response to chemotherapy and clinical outcome. Namely, many reports highlight the association between tumor infiltrating lymphocytes (TILs) and response to neoadjuvant chemotherapy in aggressive breast cancer [4,5,6,7,8,9]. TNBC and hormone receptor (HR)-negative/HER2-positive (HR-/HER2+) breast cancers show higher lymphocytic infiltrates compared to HR+ breast cancers, and increased TIL scores were associated with higher pathological complete response (pCR) rates to neoadjuvant chemotherapy [4,5,6,7,8,9,10]. Both stromal and intratumoral infiltration are associated with pCR in TNBC, while only intratumoral lymphocytes are associated with pCR in luminal cancers [11]. In particular, CD8+ cytotoxic T lymphocytes are most likely associated with a good prognosis and T regulatory (Treg) cells with an unfavorable outcome [12]. Moreover, chemotherapy induces a depletion of Treg, whereas cluster of differentiation (CD)3+ and CD8+ infiltration remain unchanged [13]. Interestingly, a significant forkhead box P3-positive (FoxP3+) cell decrease was observed in patients with a pCR, while FoxP3 remained unchanged for non-responders [13]. The tissue immune infiltrate consists not only of T lymphocytes, but also of other immune cells such as B cells, Natural Killer (NK) cells, dendritic cells, macrophages, neutrophils, eosinophils and basophils [14,15]. Ali et al. determined the relative proportions of 22 types of infiltrating immune cells in almost 11,000 breast tumors by a computational approach (CIBERSORT) by analyzing gene expression profile data [16]. They found that Tregs and macrophages were associated with worse prognosis regardless of ER status. In ER-negative tumors, those with poor immune infiltration had the poorest prognosis and those with high CD8+ T cells and activated memory T cells had a better outcome [16]. They also showed that response to neoadjuvant chemotherapy in ER-negative tumors was associated with immune infiltration by T helper (Th) cells and memory B cells [16]. In contrast, M2 macrophages were associated with a lack of pCR [16]. 

Response to chemotherapy and cancer outcomes both appear to be affected by circulating immune cells, including neutrophils, lymphocytes, and eosinophils [17,18,19]. Notably, pretreatment lymphopenia is associated with poor survival and is predictive of tumor recurrence [20]. Similarly, the neutrophil/lymphocyte ratio (NLR) and the platelet/lymphocyte ratio (PLR) were described to be significantly and independently associated with higher mortality in women with breast cancer [21]. 

Eosinophils are a subset of granulocytes characterized by their bilobed nuclei, large specific granules, and their capability to be stained by acidophilic dyes [22]. They are essentially known for their implications in host defense against parasites and in allergies [23]. Tumor-associated tissue eosinophilia (TATE) was first described several decades ago, and it is frequently observed in patients with cancer, mainly during treatment with immune checkpoint inhibitors [24,25]. Their protumoral or antitumoral roles are still controversial.

## 2. Eosinophil Biology and Functions

**Figure 1:**
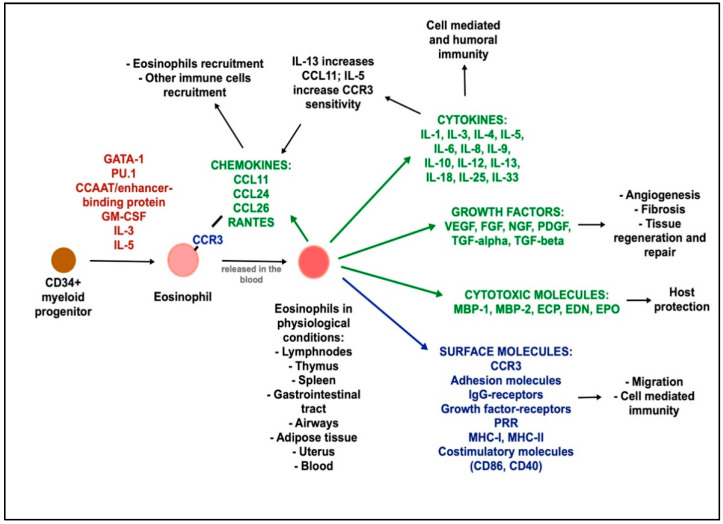
Eosinophil functions in physiological conditions. Eosinophils differentiate from a CD34+ myeloid progenitor under the stimuli from transcription factors, growth factors, and cytokines represented in red. Mature eosinophils migrate through the blood stream to several organs and tissues, being recruited due to the activity of chemokines. Eosinophils secrete several cytokines, chemokines, growth factor, and cytotoxic molecules (green), and express several surface molecules (blue), which mediate multiple functions. Abbreviations: GATA-1: GATA binding protein 1; GM-CSF: granulocyte-macrophage colony-stimulating factor; IL: interleukin; CCR3: C-C motif chemokine receptor 3; CCL: C-C motif chemokine ligand; RANTES: regulated on activation, normal T cell expressed and secreted; VEGF: vascular endothelial growth factor; FGF: fibroblast growth factor; NGF: nerve growth factor; PDGF: platelet derived growth factor; TGF: transforming growth factor; MBP: major basic protein; ECP: eosinophil cationic protein; EDN: eosinophil-derived neurotoxin; EPO: eosinophil peroxidase; Ig: immunoglobulin; PRR: pattern recognition receptor; MHC: major histocompatibility complex; CD: cluster of differentiation.

Under physiological conditions, eosinophils are essentially present in the lymph nodes, spleen, thymus, gastrointestinal tract, airways, adipose tissue, uterus, and blood, with a low concentration amounting to 1–5% of the total circulating white blood cells (Figure 1) [23]. The number of resident eosinophils is strikingly higher in the gastrointestinal tract (1.5- to 10-fold higher than in the blood) and in the lung (2-fold higher than in the blood) [23]. Eosinophils differentiate from a CD34-expressing myeloid progenitor under stimuli from several transcription factors (GATA binding protein 1 (GATA-1), PU.1, and CCAAT/enhancer-binding protein) and cytokines (granulocyte-macrophage colony-stimulating factor (GM-CSF), interleukin (IL)-3, and IL-5), and then are released into the bloodstream to migrate to different organs and tissues [22,26,27,28]. Eosinophil recruitment in different tissues is mainly dependent on eotaxin-1 C-C motif chemokine ligand (CCL11), which binds to eotaxin receptor C-C motif chemokine receptor 3 (CCR3), expressed by eosinophils, basophils, T helper, and airway epithelial cells. IL-13 enhances eotaxin-1 production, while IL-5 enhances their sensitivity to eotaxin-1 and sustains their survival [23]. Other eotaxins, such as CCL24, CCL26, and regulated on activation, normal T cell expressed and secreted (RANTES or CCL5) are also involved in eosinophil recruitment [26].

Physiological functions of eosinophils remain incompletely understood so far. They are considered to be multifunctional leukocytes, involved in immune response to infections, tissue remodeling, or other immune cell function regulations (Figure 1) [27,29]. Eosinophils contain secretory granules, containing the major basic protein 1 and 2 (MBP-1, MBP-2), the eosinophil cationic protein (ECP), the eosinophil-derived neurotoxin (EDN), and the eosinophil peroxidase (EPO), which are responsible for their cytotoxic activity [27]. They also produce many immunologic factors including cytokines (Th1- or Th2 type according to the context), lipid mediators derived from arachidonic acid, growth factors (GM-CSF, vascular endothelial growth factor (VEGF), transforming growth factor (TGF), etc.) and chemokines, to maintain their activation, promote their survival, and recruit eosinophils or other immune cells [22,26,27,30]. Moreover, eosinophils express many surface molecules including adhesion molecules, receptors for cytokines, immunoglobulin and growth factors, pattern recognition receptors (PRR), major histocompatibility complex I and II (MHC I and II), and costimulatory molecules for T lymphocytes [26]. Finally, another mechanism by which eosinophils act is by releasing mitochondrial DNA together with granule proteins to form DNA traps, which facilitate the immobilization and killing of extracellular microorganisms [31].

## 3. Eosinophils and Cancer

**Figure 2:**
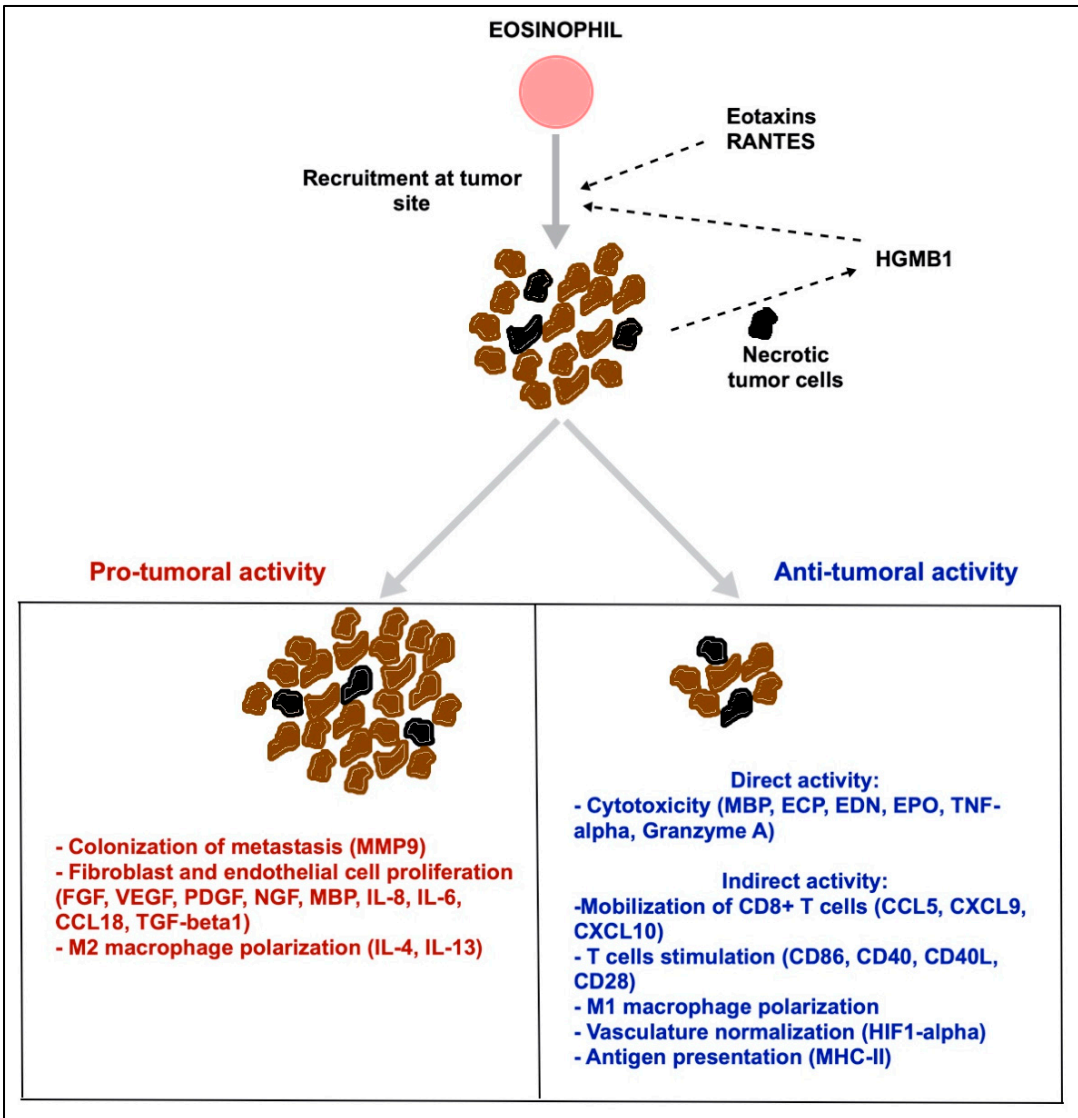
Eosinophils and cancer. Eosinophils are recruited to the tumor site through the stimuli of eotaxins and molecules released by necrotic tumor cells, such as HMGB1. At the tumor level, eosinophils may have both protumor (red) and antitumor (blue) activity, mediated by several mechanisms and molecules summarized in the figure. Viable tumor cells are represented in brown, necrotic tumor cells in black. Abbreviations: RANTES: regulated on activation, normal T cell expressed and secreted; HMGB1: high mobility group box 1; FGF: fibroblast growth factor; VEGF: vascular endothelial growth factor; PDGF: platelet derived growth factor; NGF: nerve growth factor; MBP: major basic protein, IL: interleukin; CCL: C-C motif chemokine ligand; TGF: transforming growth factor; ECP: eosinophil cationic protein; EDN: eosinophil-derived neurotoxin; EPO: eosinophil peroxidase; TNF: tumor necrosis factor; CXCL: C-X-C motif chemokine ligand; CD: cluster of differentiation; HIF1-alpha: hypoxia-inducible factor 1-alpha; MHC: major histocompatibility complex.

The association between eosinophils and cancer was described more than 100 years ago, and both protumoral and antitumoral activity have been described (Figure 2) [32,33]. Mechanisms behind their accumulation into the blood stream or tumor are uncertain. Eosinophils seem to be attracted into tumors by chemotactic factors, such as eotaxins, RANTES and damage-associated molecular patterns (DAMPs, e.g., high mobility group box 1 (HMGB1)), which are released by necrotic tumor cells [22,27]. Moreover, tumor cells or lymphocytes from TME may produce IL-5, GM-CSF, or IL-4 [22,27]. In most cases, the accumulation of eosinophils both in the tumor tissue or in the peripheral blood were reported to be associated with a better outcome [27]. 

The antitumor functions of eosinophils could be direct, by cytotoxicity through release of granules, or indirect, by modulating immune responses, especially by attracting CD8+ T cells [27]. A study showed that eosinophils secrete chemoattractant cytokines that guide CD8+ T cells into cancer tissue and induce normalization of the tumor vasculature [34]. In fact, they can enhance migration through the expression of chemokines, such as CCL5, C-X-C motif chemokine ligand (CXCL9), and CXCL10, and stimulate T cells, through costimulatory molecules, such as CD86, CD40, CD40L, and CD28 [27,29]. Eosinophils also act by inducing macrophage polarization into M1-like, which promotes inflammation and phagocytic functions. This macrophage polarization may also explain in part vasculature normalization, through the production of hypoxia-inducible factor 1-alpha (HIF1-α) [34]. Hollande et al. demonstrated that dipeptidyl peptidase-4 (DPP4) inhibitors enhance eosinophil migration into tumors in an IL33/eotaxin 1-dependent way, leading to eosinophil degranulation and reduced tumor growth [35]. Consistent with this observation, Kienzl et al. showed that eosinophils inhibit tumor growth via IL-33 in colorectal cancer murine models [36]. Eosinophils also act as antigen-presenting cells (APCs) for lymphocytes as they can process antigenic peptides, express MHC II molecules on their surface, and costimulatory molecules for T cells [22,27,37]. Mattes et al. showed that Th2 cells are responsible for the inhibition of metastases of cytotoxic T lymphocyte-resistant melanoma in mice, through eosinophil recruitment into the tumor and their degranulation [38]. Zheng et al. observed that cytotoxic T-lymphocyte antigen 4 (CTLA4) inhibitors increased infiltration of eosinophils into murine breast cancer models, leading to tumor vessel normalization and therapeutic efficacy [39]. Arnold et al. showed that the ablation of eosinophils in colorectal cancer models negatively affected antitumor immunity through defective CD8+ T cell responses. This tumor control by eosinophils is driven by the GM-CSF–interferon regulatory factor 5 (IRF5) axis and can be counter regulated by IL-10 [40]. Another study showed that intratumoral group 2 innate lymphoid cells (ICL2) in melanoma mouse models produce GM-CSF involved in the recruitment and activation of eosinophils, which in turn showed antitumor activity. This mechanism is negatively regulated by the PD-1 receptor, expressed by ICL2 [41]. Lai et al. demonstrated an antitumor capacity of pluripotent stem cell-derived eosinophils in cancer mouse models and their synergistic role with chimeric antigen receptor T (CAR-T) cells [42]. Cheng et al. showed that radiotherapy can increase intratumoral infiltration by eosinophils. Eosinophil depletion attenuates CD8+ T cell infiltration and decreases the efficacy of radiotherapy. In addition, stimulation of eosinophils by the administration of IL-5 increases CAR-T cell infiltration and supports the abscopal effect [43].

In contrast, eosinophils can exercise a protumoral role by modulation of the tumor microenvironment. To explain, they can promote distant metastases through secretion of metalloproteinase 9 (MMP9), and they can induce proliferation of fibroblasts and angiogenesis, releasing multiple growth factors and cytokines, such as tumor growth factor beta 1 (TGF-β1), CCL18, fibroblast growth factor (FGF), nerve growth factor (NGF), platelet-derived growth factor (PDGF), IL-8, IL-6, and VEGF [27]. Furthermore, through IL-4 and IL-13 production, eosinophils can polarize macrophages to the M2 phenotype, characterized by an immunosuppressive activity [27]. Sub-cutaneous injection of B16 melanoma cells into C57BL/6 wild type mice or in IL5^-/-^ mice (with eosinophils depleted) leads to the same tumor growth, suggesting that eosinophils are not able to directly eliminate tumor cells [44]. According to some studies, eosinophils had no role in the formation and growth of primary tumors, but they facilitated the colonization of the metastatic niche [27,29]. 

These anti- or pro-tumoral functions are influenced by other cells and other soluble factors from the tumor microenvironment [22].

## 4. Eosinophils and Breast Cancer

### 4.1. Tumor-Associated Tissue Eosinophilia (TATE)

TATE has been studied in several cancer types, showing a positive prognostic value in a recently published meta-analysis [45]. Few articles on head and neck and cervical cancer showed a worse prognosis in the presence of eosinophil infiltration [46,47,48,49,50]. In general, the results of different studies vary according to the tumor type, other immune cells in the TME or different activation signals. However, in most studies, eosinophils are associated with a good prognosis [27].

Few data attesting to tumor infiltration by eosinophils are reported in the literature for breast cancer. Samoszuk et al. observed EPO deposits within or around the tumor in 88% of breast cancer, but not in benign breast tissue [51]. In a transcriptomic analysis done through the computational algorithm CIBERSORT on about 11,000 cases, the authors observed an association between eosinophil infiltration and a better outcome in estrogen receptor (ER)-positive breast cancer patients, but no association with response to neoadjuvant chemotherapy was observed [16]. Chouliaras and colleagues also analyzed The Cancer Genome Atlas RNA sequencing data for eosinophil signatures in breast cancer specimens of 1069 patients through the CIBERSORT technique [52]. TATE was detected in 3.7% of the cases, mostly of luminal type. In TATE-positive patients, a prevalence of T-follicular helper cells and monocytes were observed compared to cancer without eosinophil infiltrations, while naïve B cells, resting mast cells, and resting CD4+ memory T cells were less represented. Moreover, a high level of mutations/neoantigens and an enrichment in proliferation-related gene expression was observed in TATE-positive cancers. TATE was associated with a trend toward improved DFS, but no association with OS was detected [52]. Grisaru-Tal and colleagues studied eosinophil infiltration by CIBERSORT in different tumor types, showing low infiltration in breast cancers, and a higher infiltration in gastrointestinal tract cancers [53]. They also studied eosinophil infiltrations by anti-EPO immunohistochemistry (IHC), showing a prevalent stromal infiltration in several cancer types, except for breast cancer, in which they observed a prevalence of intra-tumoral infiltration [53]. Stromal eosinophils were decreased in cancer with high expression of ER but were not associated with progesterone receptor (PgR) or HER2 [53]. A positive correlation was also observed between intratumoral or stromal eosinophil infiltration with tumor stage and primary tumor size, but not with tumor grade [53]. A recent study presented during the 2020 ESMO congress showed that an increase in eosinophil gene signature in tumor biopsies during immunotherapy was associated with response to treatment. Interestingly, in this study, a lower eosinophil gene signature was detected at baseline for responding patients [54]. 

### 4.2. Preclinical Studies in Breast Cancer

In an in vitro study, the authors demonstrated a cytostatic activity of ECP on several cell lines, including the breast cancer lines MDA-MB-453 and T47D [55]. In another study, the authors observed that, when co-cultured, eosinophils can infiltrate MCF7 breast cancer spheroids inducing apoptosis [56].

Several in vivo studies have also been conducted in breast cancer models, with conflicting results (Table 1). The administration of a DPP4 inhibitor in mice with subcutaneous EMT6 breast cancer cell implantation induced intratumoral accumulation of CCL11, resulting in eosinophilic chemotaxis and reduced tumor growth [35]. In a murine model with 4T1 breast cancer cell implantation, the administration of IL-33 induced the recruitment of NK cells in the lungs, by means of the production of CCL5 by eosinophils and CD8+ T cells, and NK cells activation, through the increase of ST2 expression (receptor for IL-33) [57]. A recent study showed that anti-CTLA4 treatment in orthotopically implanted EO771 and spontaneous mouse mammary tumor virus (MMTV)-PyVT breast cancer murine models induced tumor vessel normalization and increased treatment efficacy, through eosinophil infiltration [39]. Intravenous or intraperitoneal administration of IL-17E (IL-25) in a variety of xenograft tumor models, including breast cancer, showed an antitumoral activity alone or in combination with chemotherapy or immunotherapy due to the induction of eosinophil expansion, through the production of IL-5 [58]. A recent study conducted on mouse models of primary and metastatic breast cancer showed that the response to immunotherapy was lost after eosinophil depletion [54]. In a study by Lai et al., MDMA-MB-231 breast cancer transplanted mice showed tumor growth reduction after injection of pluripotent stem cell-derived eosinophils [42]. Conversely, other studies deposed to protumoral activity of eosinophils in vivo (Table 1). IL-33 administration, able to increase the eosinophil count by bone marrow stimulation, seems to accelerate tumor progression and development of metastases in breast cancer mice models through intratumoral accumulation of immunosuppressive cells (myeloid derived suppressor cells (MDSC), Tregs, and M2 macrophages), reducing the cytotoxicity and tumor infiltration of NK cells, and inducing neovascularization [59,60]. Shani et al. showed that the IL-33 gene is up-regulated in cancer-associated fibroblasts (CAFs) in lung metastases of spontaneous MMTV-PyMT breast cancer mouse models, resulting in the recruitment of inflammatory cells, including eosinophils, at the metastatic site. By targeting IL-33 in vivo, the authors showed an inhibition of metastasis and a reduction of inflammatory cell recruitment [61]. In another study, the authors demonstrated that myeloperoxidase (MPO) and EPO promote collagen deposition, fibroblast migration, and angiogenesis, leading to primary tumor growth and metastases [62].

### 4.3. Circulating Eosinophils in Breast Cancer

The role of the peripheral eosinophil count was widely studied in several cancer types, namely melanoma and lung cancer, during treatment with immunotherapy. In these patients, an association between both high baseline eosinophil count or increased count during treatment and better response to treatment or survival has been observed [63,64,65,66,67,68,69,70]. Their role in breast cancer is less known, with only a few reports studying the role of circulating eosinophils (Table 2). In a retrospective study, Gunduz et al. studied the prognostic value of different peripheral blood parameters in 62 patients with HER2-positive early and locally advanced breast cancer, treated with adjuvant trastuzumab. They found that patients with lower baseline eosinophil count showed better disease-free survival (DFS) rates [71]. Conversely, Ownby et al. observed a positive association between high baseline eosinophil count and lower recurrence rates in 419 patients with breast cancer of all subtypes and stages [19]. No details about the type of treatment received by the patients were available in this paper [19]. Authors also showed that low lymphocyte and eosinophil count were two highly associated parameters, observing that patients with low eosinophil count tended to have low lymphocyte count [19]. Recently, we conducted a retrospective analysis on a cohort of 112 TNBC and HR-/HER2+ breast cancer patients receiving neoadjuvant treatment, which was in the majority of the cases a combination of epirubicin and cyclophosphamide followed by taxanes (plus trastuzumab for HER2+ patients) [72]. We observed a positive association between baseline and post-surgery relative eosinophil count (REC) with pCR and survival [56]. We also studied the prognostic value of circulating eosinophils in a larger cohort of 930 early-stage breast cancer patients, observing a positive association between higher REC and better outcome, independent of the subtype [73]. In the subgroup analysis, a better prognosis for almost all subgroups was observed, in particular for patients not receiving chemotherapy or anti-HER2 treatment [73]. No subgroup analysis was performed based on the drugs received, which were in most cases a combination of epirubicin and cyclophosphamide followed by taxanes [73]. Moreover, an increase in relative circulating eosinophil count, although in the normal range, has been observed after surgery, with stability of eosinophil count for patients who did not experience relapse until 10 years of follow-up. Conversely eosinophil count decreased at relapse [73]. An improvement in time to treatment failure (TTF) and breast cancer-specific survival (BCSS) in patients with high relative lymphocyte count (≥17.5%) were also observed [73]. In a recently published retrospective study, no association between survival and eosinophil count was detected in 601 breast cancer patients of all subtypes [74]. Takahashi et al. showed an association between infusion reactions to trastuzumab and a low eosinophil count, while another study showed an association between a high level of immunoglobulin E and a lower risk of cardiotoxicity in patients treated with anthracyclines and trastuzumab, suggesting that eosinophils are also involved in tolerance to anticancer treatments [75,76].

The above mentioned studies are retrospective studies, in which some data that might impact the number of circulating eosinophils was not considered. For example, the use of corticosteroids, nonsteroidal anti-inflammatory drugs, sulfa drugs, and ni-trofurantoin, could increase the number of circulating eosinophils [77]. The use of concomitant medications was not investigated in the previously cited articles and therefore could constitute a bias to the results obtained. Prospective studies including the collection of these data could help to overcome this issue.

In addition, it would be interesting to investigate the role of eosinophils during treatment with immune checkpoint inhibitors. In fact, these drugs can induce eosinophilia, as previously shown in pulmonary cancers and melanomas [63,66,78]. In the studies listed above, there are no patients who received such treatments, considering that the first data on immunotherapy in breast cancer were only recently published.

### 4.4. Eosinophil-Related Cytokines in Breast Cancer

Several studies have assessed cytokine profiles in the serum of breast cancer patients, as their expression is often deregulated in cancers and could be used for measuring interactions between the immune system and the cancer [79]. Dehqanzada et al. measured levels of 22 cytokines in 36 HER2+ breast cancer patients and in 13 healthy women. Levels of cytokines, especially RANTES and eotaxins were higher in breast cancer patients [79]. They also compared levels of cytokines in patients with node negative versus node positive disease, finding a higher level of IL-13 in node positive patients [79]. Another study confirmed that a higher level of RANTES is seen in breast cancer patients than in healthy women [80]. Moreover, the plasmatic eotaxin level showed an inverse correlation with the number of positive lymph nodes [81]. Eotaxin seems also to be associated with anthracycline toxicity. In particular, a reduced plasmatic level of eotaxin-3 (CCL26) during treatment is associated with cardiotoxicity and a low basal eotaxin is inversely correlated with fatigue [82,83]. IL-5, a key mediator in eosinophil activation, was found to be higher in node positive breast cancer and was associated with HER2 expression [84]. IL-4 and IL-13, two cytokines contained in eosinophil granules and involved in the Th2 response and in macrophage M2 polarization, seem to be associated with a worse outcome in breast cancer [84,85,86,87].

### 4.5. Allergy and Breast Cancer

It has long been assumed that patients with allergic diseases may have a more efficient immunosurveillance capability [88]. Furthermore, the observation that cells involved in allergic reactions, such as eosinophils, may be correlated with a better prognosis, suggests a link between the allergic state and the risk of developing cancer. Numerous epidemiological studies have analyzed this association. While in most cancers results are controversial, more consistent are the data on brain and pancreatic neoplasms, for which we observed a lower risk of developing cancer in allergic patients, and on pulmonary neoplasms, where a higher risk of developing cancer for asthmatic patients has been observed [88].

Several studies have been published on breast cancer, some suggesting an inverse association between allergy and breast cancer, others showing non-significant results [88,89]. A recent published study by Bozek and colleagues, conducted in 11,101 breast cancer patients and 18,910 controls, showed a lower incidence of allergic diseases in cancer patients than in controls [90]. Further studies are needed to confirm this association and to analyze the underlying molecular mechanisms.

## 5. Discussion

The functions of eosinophils have long been restricted to their activity in parasitic infections and allergic diseases. More recently, several studies have shown that eosinophils also play a role in the immune response in the presence of cancer. Several studies have been conducted to assess the role of eosinophils in both tumor tissue and circulating eosinophils [19,63,64,65,66,67,68,69,70,71,72,73]. In particular, circulating eosinophils have mainly been studied during immunotherapy in solid tumors [63,64,65,66,67,68,69,70]. Less information is available regarding their role in patients treated with chemotherapy [72,73]. Although most studies showed an association between higher eosinophil count and better prognosis, the exact mechanisms by which they act remain unclear. In general, we know that they have antitumoral or protumoral functions, according to activation signals in the tumor microenvironment [27]. An analysis of eosinophil subpopulations could be useful to clarify the activity of these cells in the presence of a tumor. In fact, eosinophils are cells involved in numerous physiological and pathological processes, suggesting that the behavior of eosinophils in different situations depends on specific cell characteristics [91]. Historically, eosinophils were classified into hypodense and normodense, with the former having a greater cytotoxic capacity [92]. More recently, eosinophils have been divided into resident and inducible based on the expression of several phenotypic markers such as CD45, sialic acid-binding immunoglobulin-like lectin (Siglec) F (Siglec-F) for mice or 8 (Siglec-8) for human, IL-5 receptor alpha (IL-5Rα), CD11b, and CCR3 [93]. In particular, resident eosinophils show inhibitory activity of the immune system and are comparable to normodense eosinophils, while inducible eosinophils have a proinflammatory activity and are comparable to hypodense eosinophils [91]. Studies published by Johansonn and Metcalfe showed that several activation markers are more highly expressed by circulating eosinophils in allergic subjects [94,95]. In addition, eosinophils in the bronchoalveolar lavage showed lower expression of CCR3, IL-5Rα, and L-selectin (CD62L) than circulating eosinophils [96,97,98]. In a study published by Hansel et al., it was reported that eosinophils in sputum but not in blood from asthmatic patients expressed MHC-II [99]. However, MHC-II expression could be induced in circulating eosinophils under the stimulus of GM-CSF, IL-3, and interferon-γ (IFN-γ) [100]. Moreover, several studies suggest the existence of eosinophil subpopulations similar to T cells, expressing CD25, CD4, and CD28 [101,102,103,104]. There are no data in the literature so far regarding eosinophil subpopulations in subjects with cancer.

In this review, we have summarized all available data on eosinophils in breast cancer. In particular, although one study showed a worse prognosis in patients with HER2-positive breast cancer, other studies showed an association between higher circulating eosinophil count and better prognosis in all breast cancer subtypes or response to neoadjuvant chemotherapy in HR-/TNBC patients. In this regard, it is important to note that the sample size of the only negative study (n = 62) is significantly lower than that of the positive studies (see Table 2) [18,71,72,73]. Interestingly, two recent studies, published by our team, have shown that eosinophil count varies at different stages of the disease, with a lower count in the presence of cancer [72,73]. This suggests a direct role for eosinophils in anti-tumor responses, as well as a possible role of the neoplasm in modulating the number of circulating eosinophils. Some studies on TATE suggest a positive association between eosinophil infiltration and good prognosis in breast cancer. In this regard, literature data mainly include RNA sequencing analysis in large cohorts of breast cancer patients [16,52,53]. Considering the characteristic of eosinophils to express large amounts of ribonucleases (RNases), we question the reliability of using RNA sequencing data to assess the degree of tumor infiltration by eosinophils [105]. Furthermore, gene expression studies lose the possibility of analyzing the localization of the immune infiltrate in comparison with microscopic studies performed directly on tumor sections. Further studies in tumor tissue are desirable in order to confirm or reject the prognostic role of TATE in breast cancer and to observe the pattern of infiltration in different subtypes.

With regard to studies in mouse models, conflicting results were observed, some in favor of the anti-tumorigenic role of eosinophils and others in favor of a pro-tumorigenic role. These were conducted in heterogeneous models, in which both tumor cell implantation and eosinophil stimulation were done differently. Moreover, cytokines used to stimulate eosinophil differentiation have a pleiotropic effect, resulting in modulation of other cell lines as well. Considering the fine balance of the immune system, transgenic models of eosinophil modulation (such as GATA-1 knockout mice, IL5^-/-^, or IL5^+/+^ mice) or spontaneous tumor development would probably be more appropriate for this type of study, as well as the use of avatar mice.

Finally, in regards to cytokine/chemokine studies, it must be considered that the expression of a cytokine is unlikely to be associated with a single cell type. However, these studies may support the analysis of the TME and may be useful to identify biomarkers.

## 6. Conclusions

Several studies have shown that eosinophils are involved in the immune response to breast tumors, but the exact molecular mechanism involved is still under investigation. Indeed, modulation of eosinophils alone or in combination with other treatments could lead to a change in disease progression. In particular, the study of this cell type may be important with the advent of immunotherapy in breast cancers, in particular for the treatment of the most immunogenic subtypes such as TNBCs. For this reason, further studies aimed at analyzing the mechanistic role of eosinophils are required. Moreover, eosinophil count alone or in combination with other cell types could be used as predictive or prognostic biomarkers in breast cancer with advantages of being affordable and minimally invasive, although at this time we still need to validate this biomarker on an independent cohort and a larger number of patients.

## Figures and Tables

**Table 1:** In vivo studies investigating roles of eosinophils in breast tumors.

Experimental Model	Results	Role	Reference
Breast cancer mice model	DPP4-inhibitors induced intratumoral accumulation of CCL11, resulting in eosinophilic chemotaxis and reduced tumor growth.	Anti-tumoral	Hollande et al. [35]
Breast cancer mice model	IL33 suppress the development of metastasis via recruitment and activation of NK cells.	Anti-tumoral	Qi et al. [57]
Breast cancer mice model	Anti-CTLA4 therapy increases TATE, which correlates with tumor vessel normalization and anti-CTLA4 efficacy.	Anti-tumoral	Zheng et al. [39]
Different mice models including breast cancer	Administration of IL-17E has antitumor activity by inducing production of IL-5 and eosinophil expansion. A higher efficacy was observed when IL-17E was used in combination with other anticancer treatments.	Anti-tumoral	Benatar et al. [58]
Different mice models including breast cancer	Response to cisplatine plus immune checkpoint inhibitors was lost with concomitant depletion of eosinophils. Blood eosinophils increased during treatment in mice responding to immunotherapy.	Anti-tumoral	Voorwerk et al. [54]
Different mice models including breast cancer	Injection of eosinophils derived from pluripotent stem cells reduces the growth of MDA-MB-231 breast cancer.	Anti-tumoral	Lai et al. [42]
Breast cancer mice model	IL-33 accelerates breast cancer progression and development of lung and liver metastases by inducing neovascularisation, facilitating expansion of immune suppressor cells within tumor (MDSCs, ILCs, T regs, macrophages M2) and by diminishing antitumor NK cells activity.	Pro-tumoral	Jovanovic at al. [59]
Breast cancer mice model	IL-33 in tumor microenvironment reduces the apoptosis and sustains the survival of MDSCs and augments their immunosuppressive ability.	Pro-tumoral	Xiao et al. [60]
Breast cancer mice model	*IL-33* gene up-regulation in CAF associated with lung metastases. Inhibition of IL-33 reduces lung metastases.	Pro-tumoral	Shani et al. [61]
Breast cancer mice model	MPO and EPO increase primary tumor growth and promote metastases through promoting collagen deposition, fibroblastes migration and angiogenesis.	Pro-tumoral	Panagopoulos et al. [62]

Abbreviations: DPP4: dipeptidyl peptidase; CTLA4: cytotoxic T lymphocyte-associated protein 4; TATE: tumor-associated tissue eosinophilia; MDSC: myeloid derived suppressor cell; ILC: innate lymphoid cell; Treg: regulatory T cell; NK cell: natural killer cell; MPO: myeloperoxidase; EPO: eosinophil peroxidase; CAF: cancer associated fibroblast.

**Table 2:** Circulating eosinophil count and impact on breast cancer outcome.

Population	Number of Patients	Conclusions	Role	Reference
HER2+ breast cancer receiving adjuvant trastuzumab	62	Positive association between low baseline eosinophil count (≤70/mm³) and better disease-free survival rate.	Pro-tumoral	Gunduz et al. [71]
Breast cancer, all subtypes	419	Positive association between high baseline eosinophil count (≥55/mm³) and lower recurrence rate.	Anti-tumoral	Ownby et al. [19]
TNBC and HR-/HER2+ breast cancers receiving neoadjuvant chemotherapy	112	Positive association between baseline and post-surgery REC with pCR and survival rate. Increase in relative circulating eosinophil count after surgery, that remain stable for patients who do not experience relapse.	Anti-tumoral	Onesti et al. [72]
Breast cancer, all subtypes	930	Baseline REC ≥1.5% associated with better survival. Increase in REC after surgery, that remain stable for patients who do not experience relapse until 10 years of follow-up.	Anti-tumoral	Onesti et al. [73]
Breast cancer, all subtypes	601	No association between survival and eosinophil count.	No association	Zenan et al. [74]

Abbreviations: TNBC: triple-negative breast; REC: relative eosinophil count; pCR: pathological complete response.

## Data Availability

Not applicable.
